# Time to put down the scalpel? The role of surgery in tendinopathy

**DOI:** 10.1136/bjsports-2019-101084

**Published:** 2019-10-25

**Authors:** Neal L Millar, George A C Murrell, Paul Kirwan

**Affiliations:** 1 Institute of Infection, Immunity & Inflammation, College of Medicine, Veterinary and Life Sciences, University of Glasgow, Glasgow, UK; 2 Department of Orthopaedic Surgery, University of New South Wales—St George Campus, Sydney, New South Wales, Australia; 3 School of Physiotherapy, Royal College of Surgeons in Ireland, Dublin, Ireland; 4 Physiotherapy Department, Connolly Hospital Blanchardstown, Blanchardstown, Ireland

**Keywords:** tendinopathy, surgery, physiotherapy, load

‘*Constant attention by a good nurse may be just as important as a major operation by a surgeon*’ goes the famous yet rarely used quote from the early 20th century and is something we should pause and consider for our tendon patients. We argue that there is often a ‘silo’ approach in the management of tendinopathy based on the practitioner who first encounters the tendinopathy patient. Surgeons tend to ignore loading regimes, physiotherapists can be dismissive of surgery even when the patient is not making progress and we contend that many sports doctors use ‘novel’ treatment modalities which have a little evidence base.

This divergent approach to management in daily practice among specialties merely highlights how difficult the decision-making process in tendinopathy is for the treating healthcare professional. In many practitioners’ minds, surgery has always been the last resort of failed responders to various non-medical management. While expert opinions, guidelines and systematic reviews have attempted to guide clinicians on when surgery may be an appropriate next treatment step, there is little evidence comparing surgical versus non-surgical treatments.^1^

**What does the literature say?**

**Figure 1:**
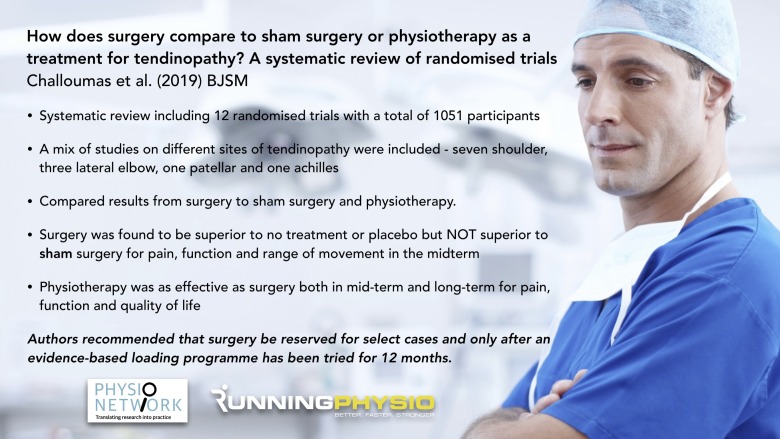
Infographic highlighting key points from systematic reviewon how surgery compares to sham surgery or physiotherapy in tendinopathy.

In view of this frustrating situation, we set out as a group of surgeons and physiotherapists to systematically review the current evidence base of surgery versus physiotherapy in an attempt to provide a balanced view that other practitioners could use to help to guide their practice in treating tendinopathy patients. In our study, published in the *BMJ Open Sport & Exercise Medicine*,^2^ we analysed 12 eligible randomised controlled trials (RCTs) in patients with various tendinopathies and found no evidence for the superiority of surgery to exercise-based therapies in patients with tendinopathy and, importantly, that outcomes after tendon loading exercises, both up to 12 months and longer-term, are as good as surgery (figure 1).

Based on these data, we advocated that healthcare professionals treating common tendinopathies should reserve surgery for selected cases and, importantly, only after a sufficiently long course (12 months) of evidenced-based loading exercise or when the patient has been unable to tolerate progressive loading and repeatedly failed to progress functional/sporting goals.

**The need for sham surgery**

Our published review highlighted the importance of sham surgery in randomised controlled surgical trials including those in tendinopathy. Compared with using a non-surgical control group, sham surgery equalises the placebo effect of surgery and gives more realistic insights into the effectiveness of the actual surgical procedure in question.^3^ The senior author (GAM) has recently completed a sham surgery study in lateral elbow tendinopathy^4^ showing no statistically significant differences between surgery and sham surgery at 6-month and 12-month postprocedure. The exact mechanisms of how surgery (corrective of sham) lead to improvement of outcomes in tendinopathy remain unclear and highlight the distinct possibility that postsurgical loading regimes may play a role and also that ‘passage of time’ is important. We, therefore, contend that further trials evaluating surgical interventions in tendinopathy should ideally include a sham surgical arm so that true surgical effect can be quantified appropriately.

So, does this mean that surgeons should put down their scalpels? We are busy clinicians and fully aware that the modern patient, from elite to recreational, is well-informed and in many instances has undertaken due diligence - they are familiar with quality research. The patient may demand treatments beyond loading early in their treatment pathways. We additionally appreciate that connecting and engaging patients in ongoing loading programmes is not straightforward and that many psychosocial aspects of common tendinopathies fail to be addressed, which may steer patients away from a loading exercise approach towards surgical/medical interventions.^5^

However, despite the apparent lure of more technologically appealing treatments, based on the current quality evidence, we recommend that clinicians persist with exercise-based loading regimes while forging strong therapeutic alliance and engagement with patients through these difficult periods. The concept that surgery is warranted when all else fails remains difficult to rationalise based on recent surgical RCTs.

We ask whether stratification of patients in future surgical trials (ie, randomising the difficult patients, when everything else has failed) is a better way to help us to identify appropriate patients that may respond to surgery rather than pragmatic ‘all-comer’ surgical RCTs. Furthermore, we can hope for the speedy arrival of true translational tendinopathy medicine^6^ where basic mechanistic studies on human tissues yield novel therapeutics for failing patients and may be a useful adjunct for clinicians moving forward. Finally, as our systematic review demonstrated, continued engagement and collaboration between surgeons and the physiotherapy/sports medicine community will build bridges in tendinopathy research and clinical care, and ultimately make our tendon patients better.
